# Early Relapse Frequency as a Potential Predictor of Generalization in Ocular Myasthenia Gravis: A Case Report

**DOI:** 10.7759/cureus.106002

**Published:** 2026-03-27

**Authors:** Jun Terai, Taiki Matsubayashi, Misako Furuki, Masato Obayashi

**Affiliations:** 1 Department of Neurology, National Hospital Organization (NHO) Disaster Medical Center, Tokyo, JPN

**Keywords:** frequent relapses, generalized myasthenia gravis, immunotherapy, ocular myasthenia gravis, predictors of generalization

## Abstract

Ocular myasthenia gravis (OMG) is characterized by symptoms confined to the extraocular muscles and progresses to generalized myasthenia gravis (GMG) in approximately half of patients, most commonly within two years of onset. Several predictors of generalization have been identified, including anti-acetylcholine receptor (AChR) antibody seropositivity, abnormal repetitive nerve stimulation (RNS) findings, older age at onset, thymoma, and greater disease severity. However, the prognostic significance of early relapse frequency in OMG remains poorly defined. We report a patient with OMG marked by early, recurrent relapses who subsequently progressed to GMG.

A 69-year-old male presented with a three-day history of diplopia. Neurological examination revealed no bulbar, respiratory, or limb weakness. Laboratory testing showed elevated anti-AChR antibody levels (12 nmol/L), and RNS demonstrated a significant decremental response in the trapezius and nasalis muscles. Imaging studies revealed no evidence of thymoma. Based on ocular symptoms and supportive serological and electrophysiological findings, a diagnosis of OMG was established. High-dose intravenous methylprednisolone led to complete resolution of symptoms. Despite maintenance therapy with low-dose oral prednisolone, the patient experienced relapses of diplopia and ptosis at two and four months after onset, each requiring additional courses of high-dose intravenous methylprednisolone. During this period, no generalized muscle weakness was observed, and anti-AChR antibody titers remained stable. Seven months after onset, the patient developed dyspnea and bulbar symptoms, accompanied by a marked increase in anti-AChR antibody levels (81 nmol/L). Clinical deterioration, abnormal respiratory parameters, and a positive edrophonium test confirmed progression to GMG. Plasma exchange resulted in rapid clinical improvement, and symptoms were subsequently controlled with prednisolone and tacrolimus.

In this case, relapses at months two and four occurred in the absence of generalized weakness and preceded generalization at month seven, suggesting that early, frequent relapses may represent a potential predictor of generalization in OMG. These findings underscore the potential importance of relapse frequency as a clinical marker and emphasize the need for treatment strategies in OMG aimed not only at symptom control but also at preventing generalization.

## Introduction

Myasthenia gravis (MG) is an autoimmune disorder characterized by autoantibodies directed against components of the neuromuscular junction, resulting in skeletal muscle weakness [1]. Ocular myasthenia gravis (OMG) presents with symptoms confined to the extraocular muscles and progresses to generalized myasthenia gravis (GMG) in approximately 50-60% of patients, most commonly within two years of diagnosis [2]. Several predictors of generalization from OMG to GMG have been identified, including anti-acetylcholine receptor (AChR) antibody seropositivity, abnormal repetitive nerve stimulation (RNS) findings, age at onset over 50 years, the presence of thymoma, and greater disease severity [3]. While established predictors of generalization are well described, dynamic disease activity markers such as relapse frequency have not been systematically evaluated.

GMG is typically managed with intensive immunotherapy, including early fast-acting treatment (EFT), to reduce disease activity and prevent further relapses, an approach that is well established [4]. In contrast, because of the lack of robust evidence and consensus guidelines [5], treatment strategies for OMG often focus primarily on symptomatic control [6].

Here, we report a patient with OMG characterized by early and frequent relapses who subsequently progressed to GMG. This case suggests that early recurrent relapse in OMG may represent a potential predictor of generalization and underscores the importance of treatment strategies aimed not only at symptom control but also at preventing progression to GMG.

## Case presentation

**Figure 1:**
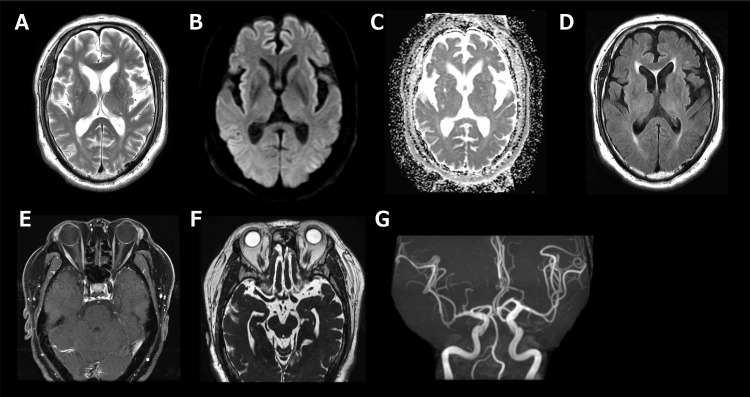
Brain MRI at admission. T2-weighted imaging (A), diffusion-weighted imaging (B), apparent diffusion coefficient (ADC) map (C), fluid-attenuated inversion recovery (FLAIR) (D), contrast-enhanced MRI (E), 3D-FIESTA (fast imaging employing steady-state acquisition) (F), and magnetic resonance angiography (G) revealed no evidence of an aneurysm or other abnormalities.

**Figure 2:**
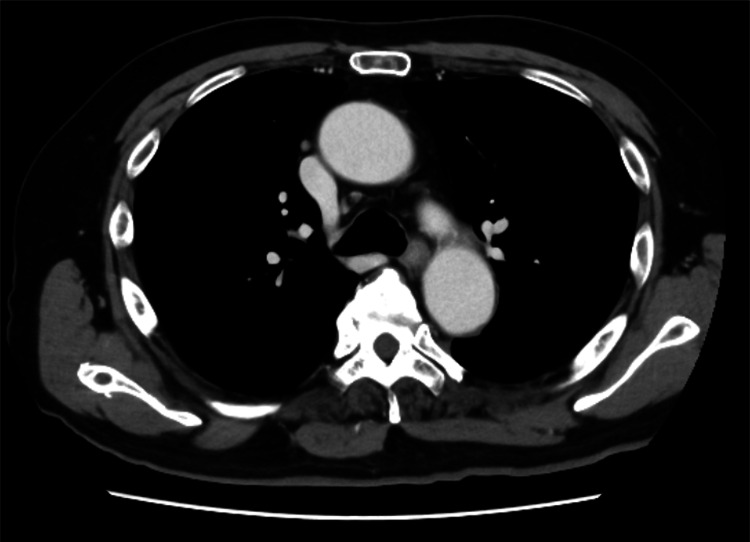
Contrast-enhanced CT of the chest. Contrast-enhanced CT of the chest revealed no abnormalities, including mediastinal masses suggestive of thymoma.

A 69-year-old male presented with a three-day history of diplopia. His medical history was notable for diabetes mellitus, with no history of antecedent infection. Neurological examination revealed restricted adduction of the left eye and bilateral diplopia on lateral gaze, without headache or ocular pain. No other neurological abnormalities were observed, including dyspnea, dysarthria, dysphagia, limb weakness, or sensory disturbances. Blood tests showed normal thyroid function, and his diabetes was well controlled (glycated hemoglobin of 6.2%). Immunological testing demonstrated elevated anti-AChR antibody levels (12 nmol/L). Anti-GQ1b immunoglobulin G antibodies, antinuclear antibodies, anti-myeloperoxidase antineutrophil cytoplasmic antibodies, anti-proteinase 3 antineutrophil cytoplasmic antibodies, and anti-SS-A/SS-B antibodies were all negative. Cerebrospinal fluid analysis showed normal white blood cell count, protein, and glucose levels. The laboratory data are summarized in the Appendices. Contrast-enhanced brain magnetic resonance imaging revealed no aneurysm or other structural abnormalities (Figure 1). Chest computed tomography showed no mediastinal masses suggestive of thymoma (Figure 2). A RNS test of the trapezius and nasalis muscles demonstrated a >10% decremental response during 3-Hz stimulation, while those of frontalis and abductor digiti minimi were negative. Based on ocular symptoms, anti-AChR antibody positivity, and abnormal RNS findings, a diagnosis of OMG was established [7]. The patient was treated with high-dose intravenous methylprednisolone (1,000 mg/day for three days), resulting in complete resolution of ocular symptoms and improvement in the Myasthenia Gravis Activities of Daily Living (MG-ADL) score from 3 (due to diplopia) to 0 [8].

Oral prednisolone (5 mg/day) was continued as maintenance therapy. However, diplopia and ptosis recurred at two and four months after onset, each requiring additional courses of high-dose intravenous methylprednisolone. After each course, symptoms resolved, and the MG-ADL score improved from 6 to 0. During this period, anti-AChR antibody levels remained stable at 12-13 nmol/L. No weakness was observed in the facial muscles other than the extraocular and orbicularis oculi muscles, nor in the bulbar, neck, respiratory, axial, or limb muscles.

Seven months after the initial presentation, the patient developed worsening diplopia and ptosis. Tacrolimus (3 mg/day) was initiated; however, dyspnea developed the following day. Peripheral oxygen saturation decreased to 85% on room air. Physical examination revealed no abnormal respiratory sounds. Neurological examination demonstrated complete limitation of ocular motility, ptosis, hypernasality, and weakness of the orbicularis oculi muscle (manual muscle testing grade 4). No limb weakness was observed, and swallowing remained intact. Anti-AChR antibody levels had increased to 81 nmol/L. Electrocardiography and chest radiography showed no significant abnormalities. An edrophonium (cholinesterase inhibitor) test demonstrated objective improvement: vital capacity increased from 59.8% to 68.5% of predicted, arterial partial pressure of oxygen increased from 64.9 mmHg to 72.8 mmHg, and eyelid fissure height improved from 3 mm to 10 mm on the left and from 3 mm to 9 mm on the right. The Quantitative Myasthenia Gravis (QMG) score [9] and MG-ADL score were 12 and 9, respectively. These findings confirmed progression to GMG.

Plasma exchange was performed six times, resulting in marked clinical improvement, particularly in dyspnea and diplopia. Vital capacity improved to 102.9% of predicted. The QMG and MG-ADL scores improved to 5 and 4, respectively. Three months after progression to GMG, symptoms remained well controlled with prednisolone (9 mg/day) and tacrolimus (3 mg/day). The clinical course and treatment timeline are summarized in Table 1.

**Table 1:** Clinical course of the patient. AChR: acetylcholine receptor; MG-ADL: Myasthenia Gravis Activities of Daily Living; IVMP: intravenous methylprednisolone; PE: plasma exchange; oPSL: oral prednisolone; PSL: prednisolone; Tac: tacrolimus; Pre: pre-treatment; Post: post-treatment.

Timeline	Clinical presentation	Anti-AChR antibody titer (nmol/L)	Treatment	MG-ADL		
				Pre		Post
Initial onset	Diplopia, ptosis	12	IVMP, oPSL	3	0	
Relapse (two months)	Diplopia, ptosis	12	IVMP, oPSL	6	0	
Relapse (four months)	Diplopia, ptosis	13	IVMP, oPSL	6	0	
Generalization (seven months)	Diplopia, ptosis, dyspnea	81	PE, oPSL	9	4	
Three months after PE	None	46	PSL, Tac	0		

## Discussion

The present case initially manifested as OMG and was characterized by three relapses within seven months. At the third relapse, the disease progressed to GMG, and plasma exchange was administered as EFT, resulting in rapid symptomatic improvement. A previous study has categorized relapses occurring within the first six months after disease onset as early relapse [10]. Therefore, this case was considered to represent early frequent relapses, as multiple relapses occurred within six months of onset.

The distinction between OMG and GMG relies on clinical distribution of muscle weakness rather than symptom duration or electrophysiological findings in facial or limb muscles [11-13]. Although the RNS test of the trapezius muscle was positive at the time of the initial diagnosis, no muscle weakness beyond the extraocular and orbicularis oculi muscles was observed until the third relapse. Therefore, we diagnosed this patient as having OMG until the third relapse, when respiratory weakness emerged. In this case, frequent early relapses preceded generalization despite the absence of generalized weakness during the initial clinical course, suggesting that the condition represented biologically active OMG and not early GMG. Moreover, the abnormal RNS finding in a non-ocular muscle suggests the possibility of subclinical neuromuscular junction involvement beyond the ocular region, even during the OMG phase, which may have indicated biologically active disease prior to generalization. This temporal pattern suggests that relapse frequency may reflect underlying immunological instability and disease activity rather than misclassification or inadequate treatment. It also highlights the potential need for earlier and more aggressive immunotherapy in selected patients.

Various predictors of conversion from OMG to GMG have been reported [3,14-16]. In the present case, the patient exhibited several established risk factors, including anti-AChR antibody seropositivity, abnormal RNS findings, and age over 50 years at onset, indicating a high risk of generalization. To the best of our knowledge, however, the prognostic significance of early relapse frequency in OMG remains poorly defined. Furthermore, the pathophysiological mechanisms underlying relapse in MG and conversion from OMG to GMG remain incompletely understood [10]. Polymorphisms of the β2-adrenergic receptor have been proposed as a potential shared risk factor for these processes [10]. Therefore, the frequent relapses observed in this patient may represent an additional clinical indicator of susceptibility to generalization. Larger studies are warranted to clarify the relationship between relapse patterns in OMG and subsequent progression to GMG.

In MG, the relationship between anti-AChR antibody titers and disease activity remains controversial. Some studies have suggested that longitudinal changes in antibody levels correlate with clinical severity [17,18], whereas others have reported inconsistent findings [19,20]. In this patient, antibody levels increased markedly from 12 to 81 nmol/L in parallel with clinical conversion from OMG to GMG, temporally associated with clinical worsening [21]. During the OMG phase, antibody levels did not decline, and frequent relapses occurred despite repeated courses of high-dose intravenous methylprednisolone combined with low-dose oral prednisolone. These findings further underscore the aggressive disease activity in this case.

For GMG, aggressive immunotherapy including EFT is recommended [22]. In this patient, plasma exchange rapidly improved both respiratory and ocular symptoms. In contrast, treatment strategies for OMG generally emphasize symptomatic relief rather than disease modification [6], largely because of the lack of robust evidence and consensus guidelines [5]. Pyridostigmine, an acetylcholinesterase inhibitor, is widely used for symptomatic management; when insufficient, oral corticosteroids are recommended [11,23]. More recently, early initiation of steroid pulse therapy for OMG has been reported to be effective, providing rapid symptomatic improvement and potentially improving long-term outcomes [24,25]. In the present case, high-dose intravenous methylprednisolone was administered at each relapse instead of pyridostigmine or high-dose oral corticosteroids, resulting in remission after each episode. According to Japanese guidelines [14], steroid-sparing immunosuppressive agents such as tacrolimus are considered when moderate or higher doses of oral corticosteroids are required for symptom control. However, in this case, rapid symptom control of OMG was achieved with steroid pulse therapy. Therefore, additional immunosuppressive agents were not initiated at that stage.

There is no established consensus that immunotherapy for OMG effectively prevents progression to GMG. Several meta-analyses of retrospective studies have suggested that immunotherapy, primarily with corticosteroids, may reduce the risk of generalization [26,27]. In our patient, corticosteroid therapy was initiated early, yet progression to GMG could not be prevented. This case raises the question of whether early relapse should trigger escalation of immunotherapy in high-risk OMG patients. Given the presence of multiple risk factors and a highly active disease course characterized by frequent relapses, earlier introduction of steroid-sparing immunotherapy may warrant future investigation. Therefore, prospective studies are required to determine whether immunosuppressive therapies beyond corticosteroids can prevent conversion from OMG to GMG.

## Conclusions

We report a case of OMG characterized by frequent early relapses that subsequently progressed to GMG. Early frequent relapses in OMG may represent a potential predictor of generalization. In addition, the RNS findings suggested subclinical non-ocular muscle involvement, even at the stage of OMG. In patients with highly active OMG, including the present case, early and aggressive immunotherapy, including the addition of immunosuppressive agents to oral corticosteroids, may be warranted, with the goal of not only achieving symptom control but also preventing generalization. However, further studies are needed to clarify the relationship between relapse frequency and generalization in OMG and to determine whether early aggressive immunotherapy can prevent progression to GMG.

## Appendices

**Table 2:** Laboratory parameters analyzed in the serum and CSF. CSF: cerebrospinal fluid; IgG: immunoglobulin G; AChR: acetylcholine receptor.

	Laboratory parameters	Value (units)	Reference value
Serum	White blood cells	8,700 /μL	4,000-10,000
	Neutrophils	37%	40-70
	Lymphocytes	55%	20-50
	Hemoglobin	11.3 g/dL	13.7-16.8
	Platelets	315,000/μL	150,000-350,000
	D-dimer	0.5 μg/mL	<1.0
	C-reactive protein	0.11 mg/dL	<0.5
	Erythrocyte sedimentation rate	14 mm/h	<10
	Blood urea nitrogen	20.0 mg/dL	8-20
	Creatinine	0.59 mg/dL	0.65-1.07
	Soluble interleukin-2 receptor	321 U/mL	122-496
	Carcinoembryonic antigen	2.4 ng/mL	<5.0
	Carbohydrate antigen 19-9	4.1 U/mL	<37.0
	IgG4	73.5 mg/dL	11-121
	Anti–AChR antibody	12 nmol/L	<0.2
	Anti-GQ1b IgG	<1.0 COI	<1.0
	Antinuclear antibody	<1:40	<1:40
	Anti-SS-A antibody	<0.4 U/mL	<7
	Anti-SS-B antibody	<0.4 U/mL	<7
	Myeloperoxidase anti-neutrophil cytoplasmic antibody	<0.2 U/mL	<3.5
	Proteinase 3-anti-neutrophil cytoplasmic antibody	<1.0 U/mL	<3.5
	Glycated hemoglobin	6.2%	4.9-6.0
	Free triiodothyronine 3	3.07 pg/mL	1.71-3.71
	Free triiodothyronine 4	1.12 ng/dL	0.70-1.48
	Thyroid-stimulating hormone	0.716 uIU/mL	0.35-4.940
	Vitamin B1	15 ng/mL	24-66
	Vitamin B12	262 pg/mL	233-914
	Folic acid	5.1 ng/mL	3.6-12.9
	Interferon-gamma release assay	Negative	Negative
	β-d-glucan	5.8 pg/mL	<20
	Blood bacterial culture	Negative	Negative
	Blood fungal culture	Negative	Negative
CSF	Color	Clear	-
	CSF pressure	130 mmH20	70-180
	White blood cells	2/3 μL	0-15
	Polymorphonuclear leukocyte	0/3 μL	-
	Mononuclear leukocyte	2/3 μL	-
	Protein	51.5 mg/dL	15-45
	Oligoclonal band	Negative	Negative
	IgG index	0.55	<0.8
	Bacterial culture	Negative	Negative
	Fungal culture	Negative	Negative
